# Overall Decrease in the Susceptibility of *Mycoplasma bovis* to Antimicrobials over the Past 30 Years in France

**DOI:** 10.1371/journal.pone.0087672

**Published:** 2014-02-04

**Authors:** Anne V. Gautier-Bouchardon, Séverine Ferré, Dominique Le Grand, Agnès Paoli, Emilie Gay, François Poumarat

**Affiliations:** 1 ANSES, Laboratoire de Ploufragan/Plouzané, Unité Mycoplasmologie-Bactériologie, Ploufragan, France; 2 Université Européenne de Bretagne, Rennes, France; 3 ANSES, Laboratoire de Lyon, UMR Mycoplasmoses des Ruminants, Lyon, France; 4 Université de Lyon, VetAgro Sup, UMR Mycoplasmoses des Ruminants, Marcy L’Etoile, France; 5 ANSES, Laboratoire de Lyon, Unité Epidémiologie, Lyon, France; Miami University, United States of America

## Abstract

*Mycoplasma* (*M.) bovis* is frequently implicated in respiratory diseases of young cattle worldwide. Today, to combat *M. bovis* in Europe, only antimicrobial therapy is available, but often fails, leading to important economical losses. The antimicrobial susceptibility of *M. bovis* is not covered by antimicrobial resistance surveillance networks. The objectives of this study were to identify resistances that were acquired over the last 30 years in France and to determine their prevalence within comtemporary strains. The minimum inhibition concentration (MIC) values of 12 antimicrobials, considered active on *M. bovis*, were compared, using an agar dilution method, between 27 and 46 *M. bovis* isolates respectively obtained in 1978–1979 and in 2010–2012 from 73 distinct respiratory disease outbreaks in young cattle all over France. For eight antimicrobials, resistances were proven to be acquired over the period and expressed by all contemporary strains. The increase of the MIC value that inhibited 50% of the isolates (MIC_50_) was: i) substantial for tylosin, tilmicosin, tulathromycin and spectinomycin, from 2 to >64, 2 to >128, 16 to 128 and 4 to >64 µg/mL, respectively, ii) moderate for enrofloxacin, danofloxacin, marbofloxacin and oxytetracycline, from 0.25 to 0.5, 0.25 to 0.5, 0.5 to 1, 32 to >32 µg/mL, respectively. No differences were observed for gamithromycin, tildipirosin, florfenicol and valnemulin with MIC_50_ of 128, 128, 8, <0.03 µg/mL, respectively. If referring to breakpoint MIC values published for respiratory bovine pathogens, all contemporary isolates would be intermediate *in vivo* for fluoroquinolones and resistant to macrolides, oxytetracycline, spectinomycin and florfenicol.

## Introduction

Formerly the name “mycoplasma” has commonly denoted bacteria of the class Mollicutes, nowadays it refers exclusively to members of the genus *Mycoplasma*. This genus comprises the simplest life forms that can self-replicate and includes major human and animal pathogens that cause diseases whose occurrence has long been underestimated [1]. All Mycoplasmas are cell-wall less bacteria and therefore are naturally resistant to all antimicrobial families that block cell wall synthesis (e.g. β-lactams and glycopeptides).

In cattle, *Mycoplasma (M.) bovis* causes respiratory disease, mastitis, arthritis and otitis [2]. It is now known that this mycoplasma species is frequently implicated in cases of bovine respiratory disease (BRD) in calves raised in feedlots worldwide [3]: it has been isolated in 40% of BRD outbreaks in the UK [3]; 25 to 80% in Italy [4], [5]; 25 to 54% in Israel [6]; and 25% to 90% in France [7], [8]. In these cases of BRD, *M. bovis* mostly occurs in coinfection with viruses and/or other bacteria but is often the only etiological agent in the chronic forms of BRD, which respond poorly to antimicrobials [2], [9], [10]. Today, only antimicrobials and sanitary controls are available to combat *M. bovis* infections. Commercial vaccines are only available in a few countries and their efficacy is subject to debate [11]–[13].

Assessing the susceptibility of mycoplasmas to antimicrobials is difficult. Some characteristics of these organisms, such as their slow growth, small size and complex growth media requirements are incompatible with the standard procedures used to test the susceptibility of classic bacteria to antimicrobials such as the disk diffusion method. The Clinical and Laboratory Standards Institute (CLSI) has only recently established standardised antimicrobial susceptibility tests to determine the minimal inhibitory concentrations (MIC) for human mycoplasma pathogens [14]. However, these procedures cannot be used for all mycoplasmas because nutritional requirements, metabolic capacities and fitness vary among species [14]. For veterinary mycoplasma species, recommendations to control the main sources of experimental bias were proposed in 2000 by the International Research Programme on Comparative Mycoplasmology (IRPCM) [15]. Today there is no veterinary reference strain well characterized for MICs to be shared for quality control purposes, which is a major hurdle to compare results from different studies. Moreover, the absence of established antimicrobial breakpoint concentrations for mycoplasmas makes it difficult to evaluate the likely *in vivo* therapeutic efficacy from MIC data established *in vitro*.

Several studies on the susceptibility of *M. bovis* to antimicrobials have been published [6], [16]–[25] but recent ones are scarce [23], [24] or have not been published so far [Gosney and Ayling, unpublished results; Cai *et al.*, unpublished results]. The experimental procedures used vary considerably: MIC tests were carried out using either the liquid broth microdilution method [16], [19], [20], [22], [23], [25], the solid agar dilution method [24] or the E test® [6], [21]. Measuring mycoplasma growth is difficult in liquid and solid media because broth turbidity is difficult to measure in a standardised way and colony size on agar can be microscopic. In broth, growth is measured indirectly by a color change of a pH indicator with the inclusion of a substrate, typically glucose, arginine or urea, according to the species. Because *M. bovis* does not use any of these substrates, alternative indirect assay methods have been specifically developed based on either tetrazolium reduction [16]; alamarBlue®, a color redox indicator [22], [23]; or phosphatase [6]. Growth has also been directly measured either by observing colonies on agar plates under a stereomicroscope [6], [21], [24] or by observing pellets after centrifuging the cultures [19]. The reference *M. bovis* type strain ATCC 25523 has often been used as a control [6], [15], [16], [21], [22], [24]; the large disparities in observed MIC values, from 5 to 8 two-fold dilutions for some antimicrobials, illustrates the difficulty in comparing studies carried out using different methods.

Reports, for most antimicrobials except fluoroquinolones, give MICs that are distributed over a large range of dilutions and suggest that strains greatly vary in their susceptibility, but without any clear separation of sub-populations. Comparative studies using a unique technique reduce the technical bias and prove to be more instructive. Cai *et al.* [unpublished results] showed that over a 20 year period in Canada, *M. bovis* acquired high and frequent resistance to oxytetracycline and macrolides. In Israel, isolates from indigenous cattle proved to be less susceptible to macrolides than those from imported bovines [6]. Strains isolated from mastitis are less susceptible than those isolated from BRD [23; Gosney and Ayling, unpublished results]. Thus the susceptibility pattern of *M. bovis* to antibiotics seems to have changed over the last few decades but in a heterogeneous way, varying according to the date of isolation, the geographical origin, the type of livestock production system and disease. Thereby, changes of susceptibility must be assessed, first and foremost at a local scale as well as by type of livestock production system [6].

The increase in antimicrobial resistance has become a real public health problem and in many countries there is growing pressure to control this resistance in both humans and animals. Curbing the progression of antimicrobial resistance includes setting up integrated treatments based on antimicrobial susceptibility tests and statistics from antimicrobial resistance surveillance networks for animal bacterial pathogens. However, mycoplasmas are generally not covered by these European networks [26]. Respiratory disease accounts for 20% of overall antibiotic consumption in cattle in France [27]. Given the high frequency of occurrence of *M. bovis* in BRD cases and its direct involvement in the chronic forms that are difficult to cure, mycoplasmas cannot be overlooked in the treatment of BRD and the antimicrobial susceptibility patterns of *M. bovis* must be updated and assessed at a regional level.

The objectives of the present study were to identify any evolution in antimicrobial susceptibility of *M. bovis* for the main classes of antimicrobials used to treat BRD in France by comparing strains isolated 30 years apart and then to assess the prevalence of the acquired resistances on a national level today.

## Materials and Methods

### Selection and Characteristics of M. bovis Isolates

The *M. bovis* isolates selected for this study came from the collection of the French national surveillance network of ruminants mycoplasmoses (VIGIMYC) [28]. Isolation was performed in Anses or in VIGIMYC-partner laboratories and identification was performed in Anses as previously decribed [29], [30]. Isolates were preserved lyophilised or −80°C frozen.

Only *M. bovis* isolated from BRD in young cattle were selected. Then two distinct groups were chosen according to the isolation date and the geographical origin: 27 “old” isolates collected in the 1978–1979 period from 27 distinct outbreaks in 20 French *départements* and 46 “contemporary” isolates collected between 2010 and 2012 from 46 distinct outbreaks in 29 French *départements*. In each of the two groups, half of the calves had been weaned and half had not. Likewise, for half of the isolates, only *M. bovis* had been isolated and for the other half, *M. bovis* had been isolated along with other bacteria, mainly *Mannheimia (M.) haemolytica*, *Pasteurella (P.) multocida* and *Trueperella (*formerly *Arcanobacterium) pyogenes.* In addition, 97 other *M. bovis* isolates collected between 2010 and 2012 across 33 French *départements* were tested for resistance to enrofloxacin. These isolates came from 90 BRD, three arthritis, three otitis and one mastitis outbreaks.

### Preparation of Inoculum for MIC Assays

Mycoplasma cultures were prepared in appropriate media from several colonies picked on agar plates after isolation. Cultures were frozen in multiple aliquots at −80°C in 15% (v/v) glycerol. To confirm species identity and to detect any mixtures of mycoplasma species, one aliquot was checked by membrane filtration dot-immunobinding tests [30] against ruminants’ mycoplasma species. For each isolate, three aliquots were used to determine the number of colony forming units (CFU) per mL by performing serial 10-fold dilutions in broth, plating each dilution on agar, incubating the plates and then counting colonies with a stereomicroscope. Final CFU/mL concentrations were expressed as the mean.

### Antimicrobial Agents Tested

Two groups of antimicrobials were successively tested.

First, six widely used antimicrobials (group n°1), from five antimicrobial classes that are likely to be active on mycoplasmas, were tested on 27 old and 46 contemporary isolates: enrofloxacin (fluoroquinolone), oxytetracycline (tetracycline), spectinomycin (aminocyclitol), florfenicol (amphenicol), tylosin and tilmicosin (macrolides). The enrofloxacin susceptibility was further tested on 97 additional comtemporary *M. bovis* isolates.

Then six other antimicrobials (group n°2) were tested simultaneously on 27 old and 30 of the 46 contemporary isolates used with group n°1: two fluoroquinolones (marbofloxacin and danofloxacin), three macrolides (tulathromycin, gamithromycin and tildipirosin) that are indicated for BRD and one pleuromutilin (valnemulin) indicated for porcine and poultry mycoplasmas.

Most antimicrobials were purchased from Sigma. Tulathromycin, gamithromycin and tildipirosin were provided by Zoetis (formerly Pfizer), Merial and MSD (formerly SP Intervet), respectively. For each antimicrobial agent, the same batch was used for all the assays.

### Preparation of Antimicrobial Dilutions

Antimicrobials in powdered form were weighed and dissolved according to the manufacturer’s instructions and drug purity. The stock solutions were prepared on the day of the MIC assay and the dilutions for use in individual MIC assays were made up in accordance with published CLSI procedures [31].

### Method of MIC Evaluation

MIC assays were performed using the agar dilution method according to recommendations by Waites *et al.* [14].

Commercial mycoplasma agar medium similar to modified Hayflick medium [15] and provided by Indicia Biotechnology was chosen since Indicia medium is recommended for growing ruminant and avian mycoplasmas and has given satisfactory performance with *M. bovis*. A single batch of medium without any inhibitor (antimicrobial or thallium acetate) was used for all MIC assays.

Doubling dilutions of the antimicrobial agents were incorporated into molten agar plates and 12 to 14 dilutions of each drug were tested. Then 1 µL of each strain diluted to yield 3×10^5^ to 3×10^6^ CFU/mL was spotted on the agar plates using a multipoint inoculator: 60 strains were simultaneously tested on the same plate for each antimicrobial dilution. Plates were incubated in ambient air with 5% CO_2_ at 37°C for 4 days. The MIC was read as the lowest antimicrobial concentration that prevented colony formation when the antimicrobial-free control plate demonstrated growth of approximately 30 to 300 CFU per spot of inoculum. MIC assays were repeated three times from three distinct aliquots of each strain and for each antimicrobial drug and final results were expressed as the median of the three MIC values.

Antimicrobials from each group were tested simultaneously on 27 old and 30 contemporary isolates in the same assay. Two other assays were conducted on 16 additional contemporary strains for group n°1 antimicrobials and on 97 contemporary strains for enrofloxacin.

### Quality Control Strains

Three mycoplasma strains were included as quality control strains for each assay: the *M. bovis* type strain PG45 isolated in 1962 (ATCC 25523), the *M. bovis* 1067 French field strain isolated in 1983 and proven to be pathogenic [32], and the *Mycoplasma gallisepticum* type strain ATCC 15302 that has been used as quality control strain several times before [33]. The *Staphylococcus aureus* type strain ATCC 29213, a standard for quality control for antimicrobial disk and dilution susceptibility tests for bacteria isolated from animals [31] was also tested in the same conditions (agar medium, drug dilutions and incubation) in order to validate the results obtained.

### Stastistical Analysis

To compare MIC distribution, a log2 transformation of the MIC data was first applied so that the variable became continuous. The Mann-Whitney test was then used to compare log2 (MIC) of old and contemporary strains of *M. bovis* for each antimicrobial, to test if one population had higher values than the other. The Mann-Whitney test is a non-parametric test and can be used with small samples [34]. The significance level was set to 0.05.

## Results

The procedure used in this study proved to be reproducible and accurate. The MICs of the *M. gallisepticum* ATCC 15302 strain and that of the two *M. bovis* strains (ATCC 25523 and 1067) were tested nine and six times, respectively, for each of the six antimicrobials of the group n°1. The variability of MIC values was always within one dilution of the median value. The 780 measurements of MICs were each repeated three times; in 99.5% of the cases, the observed values were within two successive dilutions and in the remaining 0.5% of the cases, within three. The MIC values obtained for *M. gallisepticum* were identical to those reported in other studies [33]. Those of *Staphyloccocus aureus* ATCC 29213 were consistent with the standard reference values established by the CLSI for antimicrobial susceptibility tests using a dilution method for bacteria isolated from animals [31]. Therefore, the Indicia agar medium used in the assays did not modify the availability of antimicrobials compared to Mueller Hilton medium used in standardised MIC tests for classic bacteria. Thus the scale of MIC values obtained in this study is comparable to that of standardised tests for classic bacteria.

**Figure 1:**
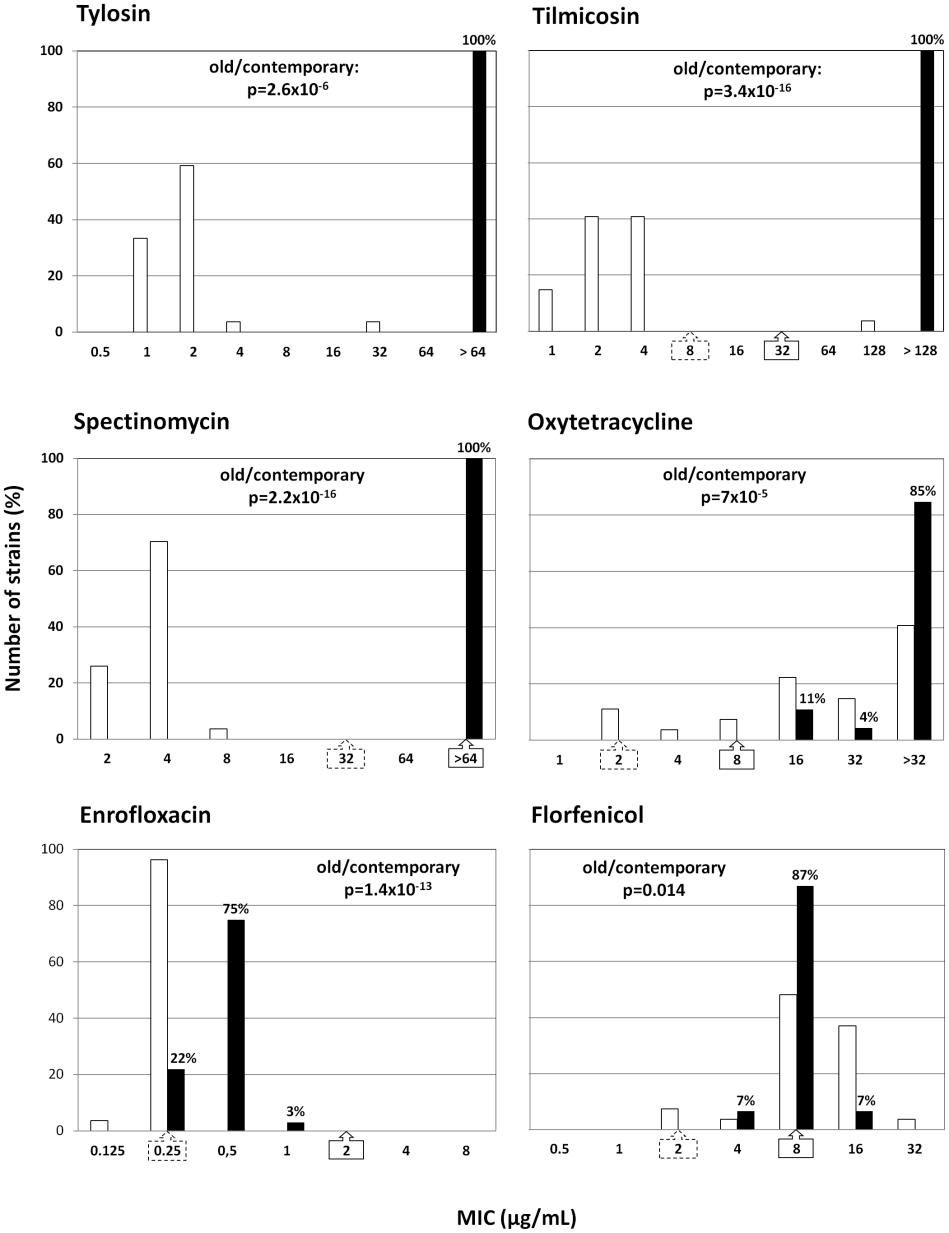
Distribution (%) of MIC values (in µg/mL) of group n°1 antimicrobials. MICs of tylosin, tilmicosin, spectinomycin, oxytetracycline and florfenicol for 27 *M. bovis* strains isolated in 1978–1979 (white bars) and 46 isolated in 2010–2012 (black bars). MICs of enrofloxacin, for 27 *M. bovis* strains isolated in 1978- 1979 (white bars) and 143 *M. bovis* strains isolated in 2010–2012 (black bars). When available, CLSI breakpoints for bovine *Pasteurellaceae* are given under the X axis: - strains with MIC values less than or equal to the dilution indicated in the dotted-line arrow are susceptible, - strains with MIC values greater than the dilution indicated in the full-line arrow are resistant, - all other strains are intermediate.

**Figure 2:**
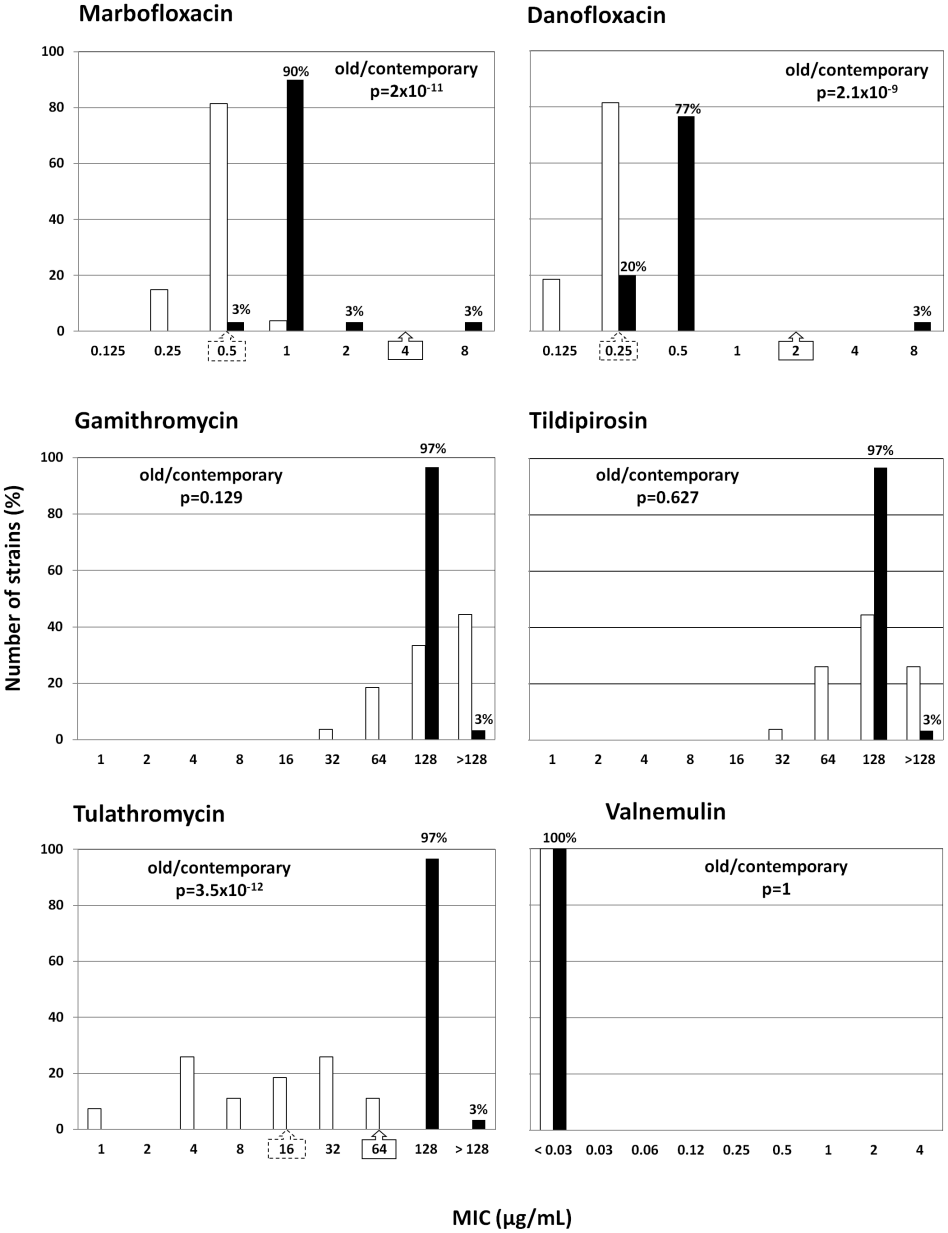
Distribution (%) of MIC values (in µg/mL) of group n°2 antimicrobials. MICs of marbofloxacin, danofloxacin, gamithromycin, tildipirosin, tulathromycin, valnemulin for 27 *M. bovis* strains isolated in 1978–1979 (white bars) and 30 *M. bovis* strains isolated in 2010–2012 (black bars). When available, CLSI breakpoints for bovine *Pasteurellaceae* are given under the X axis: - strains with MIC values less than or equal to the dilution indicated in the dotted-line arrow are susceptible, - strains with MIC values greater than the dilution indicated in the full-line arrow are resistant, all other strains are intermediate.

All MIC results are shown in Figures 1 and 2. For nine of the twelve antimicrobials tested, strain susceptibility changed significantly over time (p<0.05), with contemporary strains showing decreased susceptibility except for florfenicol for which susceptibility increased slightly. No significant change in isolates’ susceptibility was observed for gamithromycin, tildipirosin or valnemulin. The drop in susceptibility was substantial for tylosin, tilmicosin, spectinomycin and tulathromycin, with shifts in the MIC_50_ (the MIC value that inhibited 50% of the isolates) from 2 to >64, 2 to >128, 4 to >64 and 16 to 128 µg/mL, respectively. The three fluoroquinolones and oxytetracycline MIC only increased by one two-fold dilution, with shifts in the MIC_50_ from 0.25 to 0.5 µg/mL for enrofloxacin and danofloxacin, from 0.5 to 1 µg/mL for marbofloxacin and from 32 to >32 µg/mL for oxytetracycline. The screening of 97 additional contemporary *M. bovis* isolates for enrofloxacin susceptibility did not reveal any burgeoning of high level resistance in France.

The antimicrobial susceptibility pattern of contemporary *M. bovis* isolates was very homogeneous. Regardless of the antimicrobial tested, more than 77% of the strains were centred on the same MIC value or 100% showed very high MICs (spectinomycin, tylosin, tilmicosin) or very low MICs (valnemulin). Furthermore the eight resistances that have been selected over the three last decades are now observed simultaneously in 100% of *M. bovis* isolates.

The effectiveness of antimicrobials in the treatment of BRD can be estimated by comparing *in vitro* MIC values to MIC breakpoints established by the CLSI. As breakpoints were not available for veterinary mycoplasmas, MIC values were compared to breakpoints given for respiratory bovine pathogens (*Pasteurellaceae*) [31]. The mean MIC values of tylosin, tilmicosin, oxytetracycline, spectinomycin and tulathromycin for contemporary *M. bovis* strains are clearly greater than the CLSI thresholds. It is therefore very likely that these antimicrobials are not active *in vivo*. For florfenicol, 94% of strains are right at the threshold of resistance. For fluoroquinolones, all strains are classified as intermediate. Breakpoints are not available for gamithromycin and tildipirosin, but the MIC_50_ (128 µg/mL) is much greater than the maximum concentrations used for respiratory infections at therapeutic doses, 18 and 15 µg/g of lung homogenate, respectively [35], [36].

## Discussion

As with all Mollicutes, *M. bovis* is naturally resistant to β-lactams and glycopeptides, but also to polymyxins, sulfonamides, trimethoprim, nalidixic acid, rifampicin and lincomycin [37]. This study shows that *M. bovis* strains recently isolated in France (2010–2012) have become less susceptible to other antimicrobials that, until now, have been recommended or likely to be of interest for treating mycoplasmoses.

### Procedure was Repeatable and Accurate, and Sampling Adequately Chosen to Address the Objectives

The agar dilution method was used in this study because it has been recommended for *M. bovis* by the IRPCM [15]. This method has been infrequently used in previous studies because it is very labour-intensive, but it ensures a better standardisation than liquid microdilution methods. The indirect assessment of growth by the use of a substrate in broth microdilution tests is imprecise. The change in colour (of the broth) occurs gradually and requires a high titre of mycoplasmas. Measurement of *M. bovis* growth in liquid media is less standardised than for most other mycoplasma species because *M. bovis* does not utilise commonly used substrates (glucose or arginine). Furthermore, substrate oxidation kinetics for *M. bovis* can vary with strain [38], therefore generally requiring readings at several intervals. By contrast, agar dilution method can be used to test a large number of strains simultaneously on the same batch of agar with the same antimicrobial dilution (via the use of a multipoint inoculator) and growth can be directly and unambiguously assayed by direct observation of colonies under a stereomicroscope.

The unimodal or bimodal distribution of MIC values within the *M. bovis* sample obtained in the present study for all the tested antimicrobials contrasted sharply with the distributions reported in most previous studies [6], [19]–[24]. The MIC values reported for most antimicrobials in these studies, with the exception of fluoroquinolones, were scattered over a large range of dilutions. This scatter, to some extent, may be due to bias of the methods employed or to the choice of samples. Indeed the margin of error of a measurement of MIC in Mollicutes can be high, even in strictly standardised conditions, particularly for certain antimicrobials such as macrolides [14]. The procedure used in the present study was based on the agar dilution method recommended by CLSI for human mycoplasmas [14] and proved to be highly repeatable and accurate. Sampling heterogeneity can also cause scattered MIC values because the susceptibility pattern of *M. bovis* strains varies considerably with region, isolation date, disease and production practices [6, 23, Gosney and Ayling, unpublished results]. To limit sampling bias as much as possible, we chose to compare strains that were clearly different with respect to their date of isolation, but comparable in terms of country origin, disease, animal age class and organ.

### The Susceptibility of *M. bovis* to Antimicrobials Dramatically Decreased over the Last Decades

Over the 30-year interval between isolate samplings, the susceptibility of *M. bovis* decreased significantly for eight antimicrobials of tetracycline, fluoroquinolone, aminocyclitol, and macrolide families that are considered to be active on mycoplasmas.

All MIC values of spectinomycin and oxytetracycline towards contemporary French strains became very high. The wide distribution of MIC values for oxytetracycline in the old strains population suggests that less susceptible strains had arisen by 1978–79 for this antimicrobial, which was placed on the market in the 1950s. Such steep susceptibility decreases for these antimicrobials were not observed in other studies. Several recent studies show that the MIC_90_ values for spectinomycin in Britain, the USA, Canada and Japan are still less than 32 µg/mL [22, 24; Gosney and Ayling, unpublished results; Cai *et al.*, unpublished results] and less than 16 µg/mL for oxytetracycline in Britain, Israel, and the USA [6, 22, Gosney and Ayling, unpublished results].

For first-generation macrolides (tylosin, tilmicosin), the same steep decrease in susceptibility as in France was described worldwide, first in the UK in the 1990s [19] and then in USA, Israel, Canada and Japan [6, 22, 24, Cai *et al.*, unpublished results]. For the new-generation macrolides, gamithromycin and tildipirosin, MICs were high for both old and contemporary strains, pointing to a putative natural resistance as observed for erythromycin. In fact, these high values cannot be attributed to a lack of availability of the antimicrobials in the agar medium as MIC values obtained against the *M. gallisepticum* ATCC 15302 strain were very low (<0.03 and 0.25 for gamithromycin and tildipirosin, respectively). For tulathromycin, old strains were significantly more susceptible, but several old strains with intermediate MIC level were observed before tulathromycin was placed on the market (European agreement obtained in 2004). As resistance for tylosin and tilmicosin in the period before 1980 was observed for only one isolate, the occurrence of these less susceptible old strains could be better explained by naturally resistant variants than a putative cross-resistance with first generation macrolides. Interestingly, two distinct populations were also observed in a study on the action of tulathromycin based on 53 European *M. bovis* strains isolated between 1980–2002, with MIC_50_ of 0.25 and >64 µg/mL, respectively [39].

The decrease in susceptibility with respect to fluoroquinolones is significant but low (only one dilution). Although there has been a recent report of *M. bovis* mastitis strains with very high MICs for enrofloxacin [Gosney and Ayling, unpublished results], further screening of 97 additional contemporary isolates did not reveal any burgeoning resistance of high level in France.

For florfenicol, only a little but significant increase of susceptibility was observed between old and contemporary strains. The MIC_50_ remained constant at 8 µg/mL. However, seven old strains were more susceptible with MICs of 2 µg/mL. MIC_90_ values of less than 2 µg/mL have also been reported in a recent study [40]. This suggests that the susceptibility of French old strains may have already changed in 1978–79 with respect to the natural level of susceptibility. This early change in susceptibility may be a consequence of the massive use of chloramphenicol – which has a common cross-resistance mechanism with florfenicol [41] – between 1950 and 1967 (the year it was taken off the market).

### The Prevalence of Multi-resistant *M. bovis* Strains is Very High now in France

In this study, resistances proved to be acquired over the last 30 years for eight antimicrobials, simultaneously affected all the contemporary strains. This 100% prevalence of multi-resistant strains was obtained on a large and diversified sample of strains and may be close to the current national prevalence. That is exceptional in terms of bacterial resistance. Similar findings have been recently reported in UK [Gosney and Ayling, unpublished results] and Canada [Cai *et al.*, unpublished results] but to a lesser extent with respect to number of antimicrobial agents associated with resistance and level of prevalence. This phenomenon cannot be attributed to a simple sampling bias, because the 46 tested strains were isolated from 46 different outbreaks that occurred in 25 *départements* across France over a period of two years. The probability of a direct link between all these outbreaks is therefore very low. However the spread of a unique clone of *M. bovis* across all of France could explain a unique resistance pattern for all isolates. The spread of enrofloxacin-resistant strains documented in Israel was not clonal in nature [42]. Sub-typing studies on *M. bovis* conducted locally in feedlots in France [43] and at the national level in other countries [44]–[47], have concluded that the genetic diversity in contemporary mycoplasma outbreaks is usually high, but with a clonal origin in Austria [48]. This hypothesis is currently being explored by our laboratory as far as French isolates are concerned.

### The Pressure and Strategy of Antimicrobial Therapy could be Major Selective Factors of Resistance

The pressure of antimicrobial therapy could be a major selective factor in *M. bovis*, as is the case for other mycoplasma species. Experimental data based on *in vitro* cultures in the presence of antimicrobials confirms that mycoplasmas can very rapidly acquire resistance to antimicrobials. High levels of resistance to macrolides and enrofloxacin in *M. gallisepticum*, *M. synoviae* and *M. iowae* have been obtained in only a few passages [33]. During experimental infections in swine and chicken, infected by *M. hyopneumoniae* and *M. synoviae*, respectively, clones resistant to enrofloxacin have been isolated after only two treatments at therapeutic doses and were directly linked to a point mutation in the “quinolone-resistance determining region” of a topoisomerase gene [49], [50]. The rapidity with which resistance is selected may be related to the high mutation rate in mycoplasmas, likely due to a deficit in genetic information dedicated to DNA repair in mycoplasma genomes [51]. Horizontal gene transfer is also an essential factor in the spread of resistance in other bacteria. In mycoplasmas, the possibility of frequent and large transfers that had been predicted earlier from *in silico* data [52] was very recently demonstrated *in vitro* [53]. However to date the only resistance genes known in mycoplasmas to be carried on a mobile genetic element (conjugative transposon) are the *tet*M gene that encode a tetracycline resistance [37].

The strategy of antibiotherapy could also be a predominant factor in the spread of resistant strains. In *M. pneumoniae*, macrolide-resistant strains, unknown before 2000, now represent 10%, 40% and 80% of strains isolated in Europe, Japan, and China, respectively, after outbreaks of worldwide epidemics in 2010–2011 [54]. The pronounced differences in incidence among countries may be explained by more extensive macrolide use in Asia for pneumonia treatment [54]. It may be the same for *M. bovis*. BRD is a multifactorial disease in which several agents occur simultaneously or sequentially, including viruses, mycoplasmas and classic bacteria, mainly *Pasteurellaceae*. The susceptibility of *Pasteurellaceae* to antimicrobials is closely monitored in France (RESAPATH network) [55]. Based on statistics from the network, first-line treatments recommended for BRD today in France target only these *Pasteurellaceae* and do not take into account mycoplasmas. Thus, antimicrobial drugs that are often inappropriate for mycoplasmas, such as β-lactams, that are very active on *Pasteurellaceae*, are frequently used. It is likely that the administered antimicrobial treatments, by eliminating other competing bacteria, actually promote mycoplasmosis and lead to the more chronic forms described for *M. bovis* [2] and therefore to additional antimicrobial treatments. Furthermore, the absence of any systematic diagnosis for *M. bovis* and the lack of recent statistical data on its recent susceptibility pattern may result in unsuitable treatment leading to persistence and selection of even more resistant strains. In support of this hypothesis, high levels of resistance have also been found in *M. bovirhinis*, a frequent but non-pathogenic resident of the respiratory tract [24].

### Most Currently Used Antimicrobials would now be Inactive or Weakly Active on *M. bovis* Diseases but Further Investigations are Needed to Confirm

Choosing first-line active drugs to fight respiratory infections *in vivo* is the key for this type of epidemiological situation. Since there is no standard breakpoint for *M. bovis*, several authors [6], [21]–[23], [42] used breakpoints based on epidemiological and pharmacokinetic criteria established by the CLSI for bovine *Pasteurellaceae* [31]. These bacteria occur in the same disease (i.e. BRD) and at the same level (extra-cellular and in deep lung) as *M. bovis*. The conditions for reaching therapeutic concentrations *in situ* are therefore theoretically equivalent. Moreover, the scale of MIC values obtained in this study has proven comparable to that of standardised tests for classic bacteria.

Refering to CLSI breakpoints when available, 100% of contemporary *M. bovis* strains would not be inhibited *in vivo* by any antimicrobial tested in the study, except fluoroquinolones when a high dosage can be used. Very low MIC values (<0.03 µg/mL) obtained for valnemulin are therapeutically interesting, but this antimicrobial is currently used only in swine and poultry and is somewhat toxic in various animal species. This antimicrobial, administered by the oral route, has experimentally proven to be effective in calves infected with *M. bovis* strains with a MIC of 0.0625 µg/mL [56].

Conclusions on the likely therapeutic effectiveness of these antimicrobials must be taken with caution: results on *in vivo* and *in vitro* susceptibility are not always concordant. Some treatments seem to be effective in experimental infection models despite the use of strains with high MIC values [3]. Accordingly, tulathromycin has proven effective despite a MIC of >64 µg/mL on the assayed *M. bovis* strain [39]. The efficacy of gamithromycin on a *M. bovis* strain has also been proven experimentally in an infection model [57]. In contrast, therapeutic failures have been observed experimentally with *M. bovis*, *M. hyopneumoniae* and *M. synoviae* [49], [50], [58] despite the high susceptibility of inoculated strains. Other factors could indirectly affect the efficacy of a treatment, such as the production of biofilms by mycoplasmas [59] or systematic reinfection after treatment [47].

Finally the frequency of resistant strains in this study may be overestimated compared to that of currently circulating strains. The strains tested in this study came from diagnostic laboratories that were usually called after treatment failures. Antibio-surveillance networks are based on the same type of reporting and the possible overestimation does not lead to erroneous public/animal health measures. However, the rapidity of adaptation in mycoplasmas may exacerbate this bias in estimation. For instance, *M. agalactiae* isolates obtained from goats herds with clinical symptoms of *M. agalactiae* mastitis featured higher MIC values for many antimicrobials compared with isolates from asymptomatic animals [60].

### Conclusion

It is now generally accepted that *M. bovis* is frequently involved in bovine diseases such as mastitis, arthritis, otitis media, and particularly respiratory disease worldwide. The rapid decrease in susceptibility of this pathogen to antimicrobials is of high concern, particularly because it causes over-consumption of antimicrobials including those that are critical for human health. It is now important to set up systematic screening of *M. bovis*, adapt BRD treatment strategies accordingly, monitor the overall susceptibility of mycoplasmas to potentially active antimicrobials and determine their actual therapeutic activity *in vivo*. However, given the current situation and the speed at which resistance appears to be selected in mycoplasmas, alternative control measures must be rapidly set up, such as preventive health measures and the development of vaccines.
